# Delayed onset of anterior spinal artery syndrome caused by retropulsed bone fragment after kyphoplasty: Case report and literature review

**DOI:** 10.1016/j.inpm.2023.100264

**Published:** 2023-06-24

**Authors:** Royce Copeland, Colton Reeh, Ryan D'Souza, Eliana Ege, Daniel Briggi, Christian Vangeison

**Affiliations:** a H. Ben Taub Department of Physical Medicine and Rehabilitation, Baylor College of Medicine, Houston, TX, USA; b Department of Anesthesiology and Perioperative Medicine, Mayo Clinic, Rochester, MN, USA

**Keywords:** Kyphoplasty, Anterior spinal artery syndrome, Anterior cord syndrome, Spinal cord injury, Acute myelopathy, Bone fragmentation

## Abstract

**Background:**

Percutaneous balloon kyphoplasty is a minimally invasive technique to treat refractory symptomatic osteoporotic vertebral compression fractures. A rare complication called anterior spinal artery syndrome has been documented several times in the literature after the procedure from cement embolism; however, the authors report an unusual case of anterior spinal artery syndrome following kyphoplasty through retropulsion of bone fragmentation.

**Case presentation:**

An 83-year-old male was admitted to an acute care hospital for severe low thoracic back pain without neurological symptoms. Computed tomography imaging showed T8 vertebral body compression fracture with 75% height loss. Kyphoplasty was performed four days after the admission without complications. On day nine postoperatively, the patient developed acute onset paraparesis of the lower extremities dissociated sensory deficits involving bilateral loss of temperature and pain, but preserved proprioception and vibratory sense. Magnetic resonance imaging of the spine revealed T2 intramedullary hyperintensity spanning from T7-9 and retropulsion of the bone fragments from a refracture of the T8 vertebral body.

**Conclusion:**

This study highlights a rare complication from a standard pain procedure through an unusual mechanism of injury. Clinicians who suspect acute myelopathy following vertebral augmentation procedures should obtain a computed tomography angiogram to identify a potential occluded vessel. If negative, individuals should proceed to magnetic resonance imaging to rule out retropulsion of bone fragmentation into the spinal cord.

## 1 Introduction

Percutaneous balloon kyphoplasty is a minimally invasive technique to treat refractory symptomatic OVCF and misalignment of the vertebral column. This procedure requires the placement of a pressurized balloon into the vertebral body with an inflation/deflation sequence to create a cavity before injecting the bone cement, usually polymethylmethacrylate (PMMA), to augment the deficient bone [1]. Percutaneous balloon kyphoplasty has been utilized for the last several decades as an effective intervention for OVCF, but serious complications have been reported with this procedure. The most common complication is bone cement leakage, which is often asymptomatic but can have detrimental neurological complications if the cement extravasates into a blood vessel or spinal cord [1,2].

A rare complication called anterior spinal artery syndrome (ASAS) most commonly occurs as a complication of aortic surgery from prolonged cross-clamping of the aorta, but there are many other etiologies correlated with occlusion or hypoperfusion of the anterior spinal artery (ASA) [3,4]. There have been rare reports in the literature of ASAS following kyphoplasty with cement entering the circulatory system, leading to infarction of the spinal cord from inadequate blood supply of the surrounding arteries. Although vascular pathology is the most common etiology responsible for ASAS, we report another possible mechanism for this rare complication: mechanical herniation of bone fragments from the vertebral body. Below, we report the rare case of ASAS that occurred nine days following a kyphoplasty procedure with retropulsion of bone fragments from the T8 vertebral body into the spinal cord. Further, we perform a literature review of prior cases of ASAS following vertebral augmentation procedures (kyphoplasty and vertebroplasty).

### 1.1 Materials and methods

A literature search was performed in the PubMed database using the following search terms: (“anterior spinal artery syndrome”) OR (“anterior spinal cord syndrome”) OR (“anterior cord syndrome”) AND (kyphoplasty OR vertebroplasty) from 1990 to the present. Three cases of ASAS following kyphoplasty or vertebroplasty were identified and described in Table 1. Written informed consent was obtained from the patient in this current case report. Institutional approval was waived.

**Table 1:** Overview of literature on ASAS following vertebral augmentation.

Author & Year	Age	Sex	Procedure	Onset of Symptoms	Site of Injury	Mechanism of Injury	Imaging Modalities	Subsequent Interventions
Tsai et al. 2010	63	F	Vertebroplasty at T9 & T10	Immediate after post-operative	T10	Cement embolism in the paravertebral vessels, left intercostal (T10), and anterior spinal artery (T10-11).	CT without contrast	Intravenous methylprednisolone and no surgical intervention
Yazbeck et al. 2011	20	M	Vertebroplasty T8, L1	Immediate after post-operative	T10	Cement embolism in the anterior spinal artery and left intercostal artery (T10)	CT without contrast	Intravenous dexamethasone and no surgical intervention
Bredow et al. 2014	75	F	Kyphoplasty L1	Immediate after post-operative	L1	Not identified	MRI	Low molecular heparin bolus and no surgical intervention
Copeland et al. 2022	83	M	Kyphoplasty T8	Delayed (9 days post-operative)	T8	Retropulsion of the T8 vertebral body	MRI without contrast	Intravenous dexamethasone and no surgical intervention

## 2 Case Presentation

**Fig. 1:**
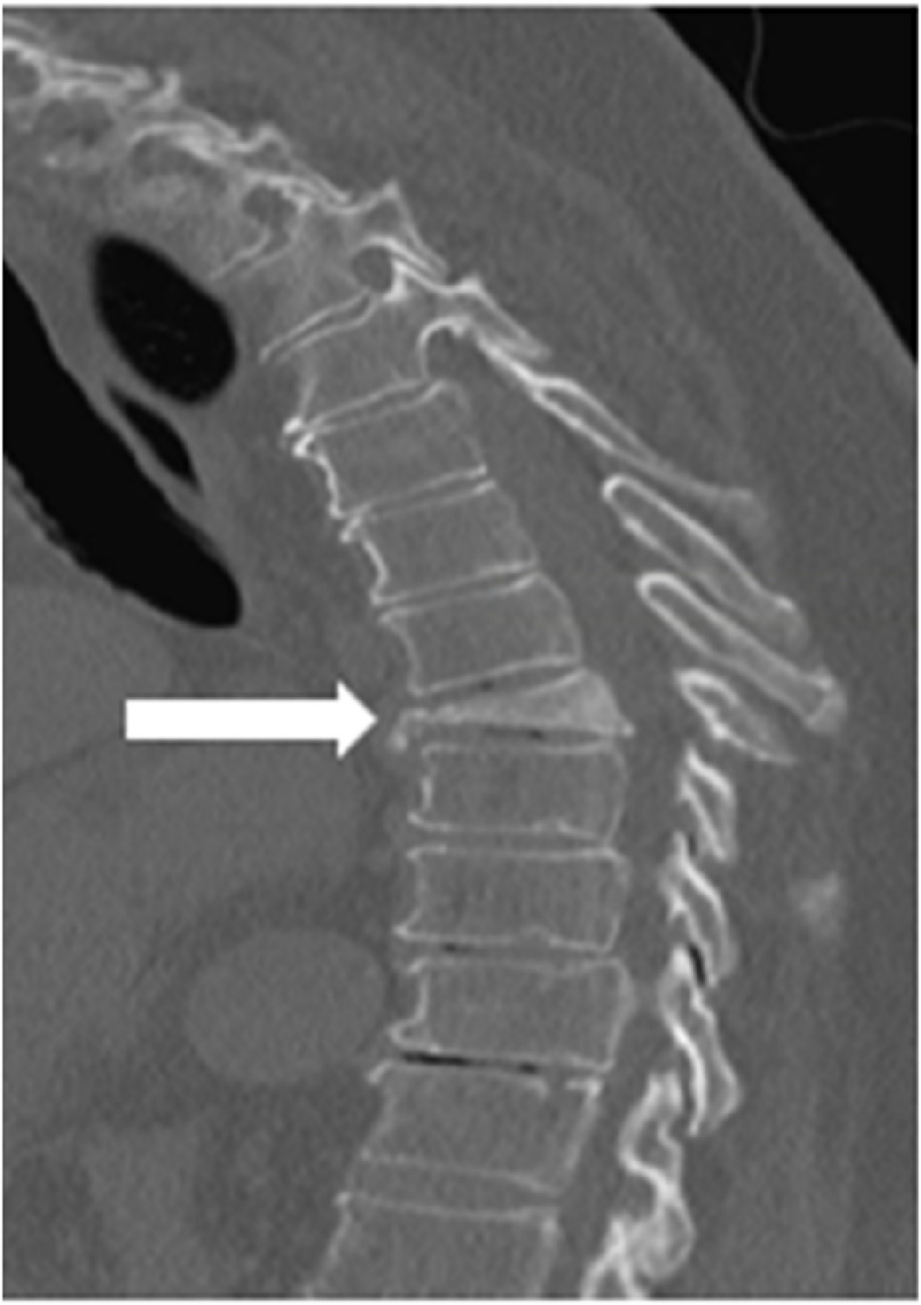
Sagittal CT reveals T8 compression fracture with 75% height loss.

**Fig. 2:**
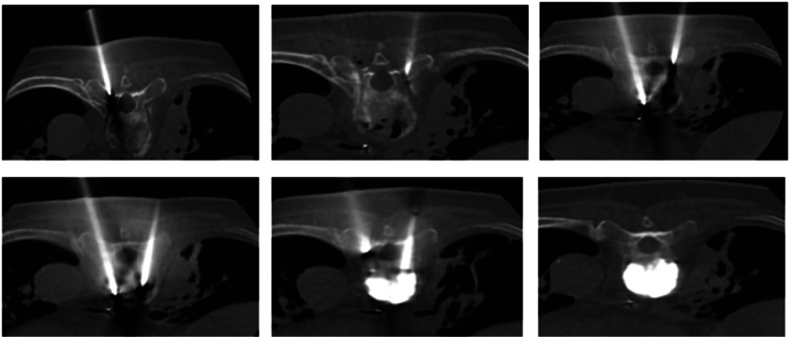
Axial CT imaging of bilateral transpedicular approach.

An 83-year-old male with a significant past medical history of prostate cancer (status post radical prostatectomy ten years prior), chronic kidney disease stage, and osteoporosis (on bisphosphate medication) presented to the emergency department for excruciating axial and mechanical mid to low back pain without lower extremity radicular symptoms. The patient reported that he suffered a previously untreated injury to his back while working on his car approximately six weeks prior. On admission, the clinical exam revealed five out of five motor strength throughout the upper and lower extremities. Light touch and pinprick sensation were grossly intact with no focal deficits in his extremities. Deep tendon reflexes were normal, and Babinski reflexes were downgoing. The only deficit appreciated on the physical exam was a limited range of motion throughout the thoracic and lumbar spine secondary to pain. Initial imaging, including an x-ray and computed tomography (CT) scan, showed the presence of a T8 vertebral body compression fracture with 75% loss of height and mild bony retropulsion without compression of the spinal canal cord [Fig. 1]. Four days after admission, the patient underwent percutaneous kyphoplasty with interventional radiology using a bilateral transpedicular approach. 8.5 mLs of PMMA was injected inside the T8 vertebral body with post-fluoroscopy images showing homogenous PMMA distribution within the vertebral body. No cement leaks were identified. [Fig. 2].

**Fig. 3:**
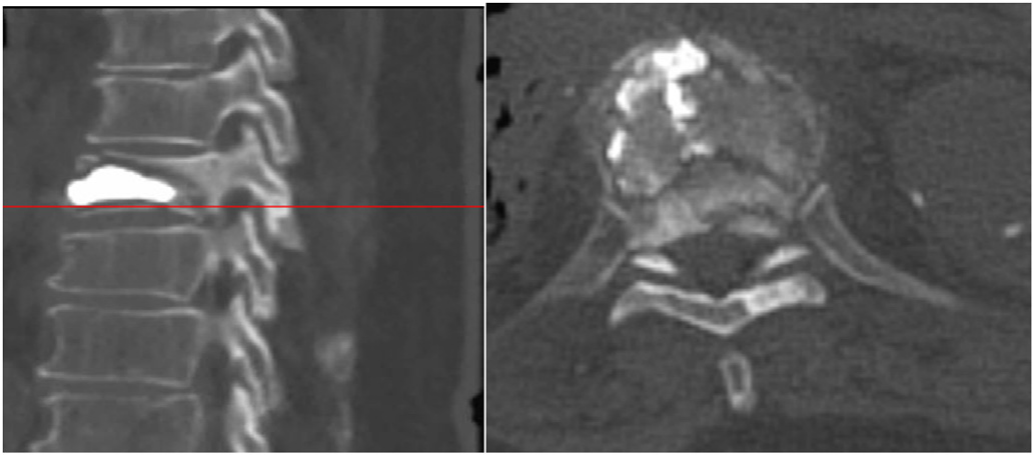
CT without contrast T8 vertebral body fracture with retropulsion of bone fragments contributing to moderate to severe canal stenosis.

**Fig. 4:**
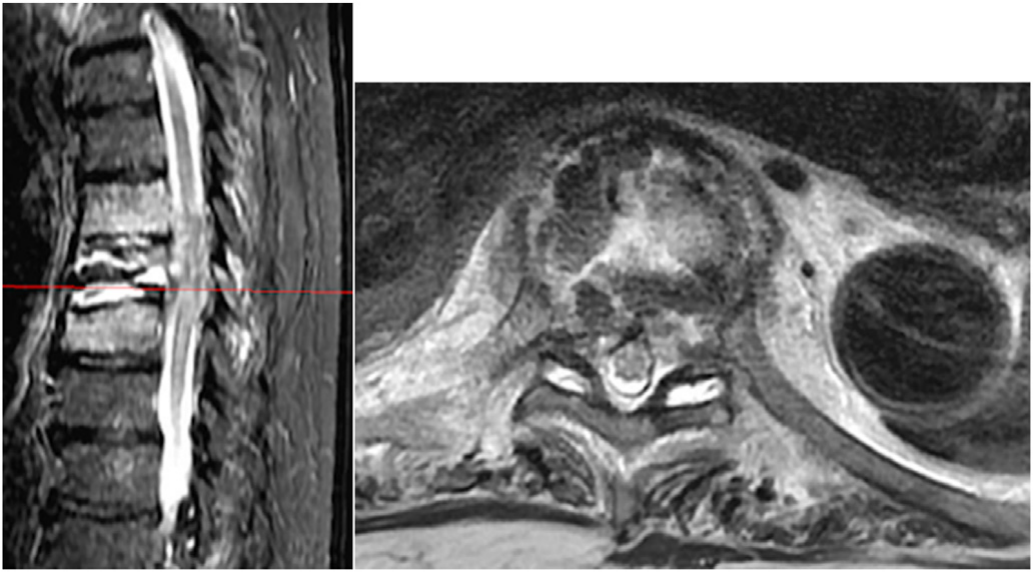
Magnetic resonance imaging (MRI) STIR of the thoracic and lumbar spine revealed abnormal T2 intramedullary hyperintensity spanning the T7-9 level with moderate to severe central canal stenosis and a 5 mm retropulsion of the T8 bone fragments disrupting posterior vertebral rim.

For the first seven days after the procedure, the patient worked with physical therapy at a functional level of minimal assistance with activities and ambulation. On the eighth postoperative day, the patient experienced a recurrence of excruciating back pain similar to his initial presentation. One day later, he developed acute-onset paraparesis of the lower extremities as well as loss of pain and temperature sensation with preservation of proprioception and vibratory sense. Deep tendon reflexes were brisk and symmetric. Hoffman sign were present bilateral. An emergent CT spine was performed without contrast due to a severe acute kidney injury showing evidence of T8 vertebral body refracture with prior vertebral augmentation and retropulsion contributing to moderate to severe compression with recommendations for more advanced imaging with MRI [Fig. 3]. Magnetic resonance imaging (MRI) of the thoracic and lumbar spine revealed abnormal T2 intramedullary hyperintensity spanning the T7-9 level with moderate to severe central canal stenosis and a 5 mm retropulsion of the T8 bone fragments [Fig. 4]. A new leukocytosis triggered an extensive workup, including blood cultures, urine cultures, erythrocyte sedimentation rate, c-reactive protein, thyroid-stimulating hormone level, and tests to detect cytomegalovirus, Ebstein-Barr virus, Lyme disease; all tests were found to be within normal limits or negative. Abscess or phlegmon was ruled out on MRI. The patient was started on a three-day course of oral dexamethasone 4 mg every 6 ​h and per neurosurgery, the patient declined posterior spinal decompression surgery. The patient was diagnosed with T7 American Spinal Injury Association Impairment Scale (AIS) C incomplete paraplegia secondary to anterior spinal cord ischemia from retropulsion of the bone fragments [Table 2].

**Table 2:** 

ASIA Impairment Scale (AIS) Classification	
A ​= ​Complete	No sensory or motor function is preserved in the sacral segments S4-5
B = Sensory Incomplete	Sensory but not motor function is preserved below the neurological level and includes the sacral segments S4-5 AND no motor function preserved more than three levels below the motor level on either side body
C = Motor Incomplete	Motor function is preserved for voluntary anal contraction OR the patient meets the criteria for sensory incomplete status, and has some sparing of motor function more than three levels below the ipsilateral motor level on either side of the body. For AIS C – less than half of key muscle functions below the single NLI have a muscle grade ≥3.
D ​= ​Motor Incomplete.	Motor incomplete status as defined above, with at least half (half or more) of key muscle functions below the single neurological level of injury having a muscle grade ≥3.
E ​= ​Normal.	If sensation and motor function as tested with the ISNCSCI are graded as normal in all segments, and the patient had prior deficits, then the AIS grade is E. Someone without an initial SCI does not receive an AIS grade.

**Fig. 5:**
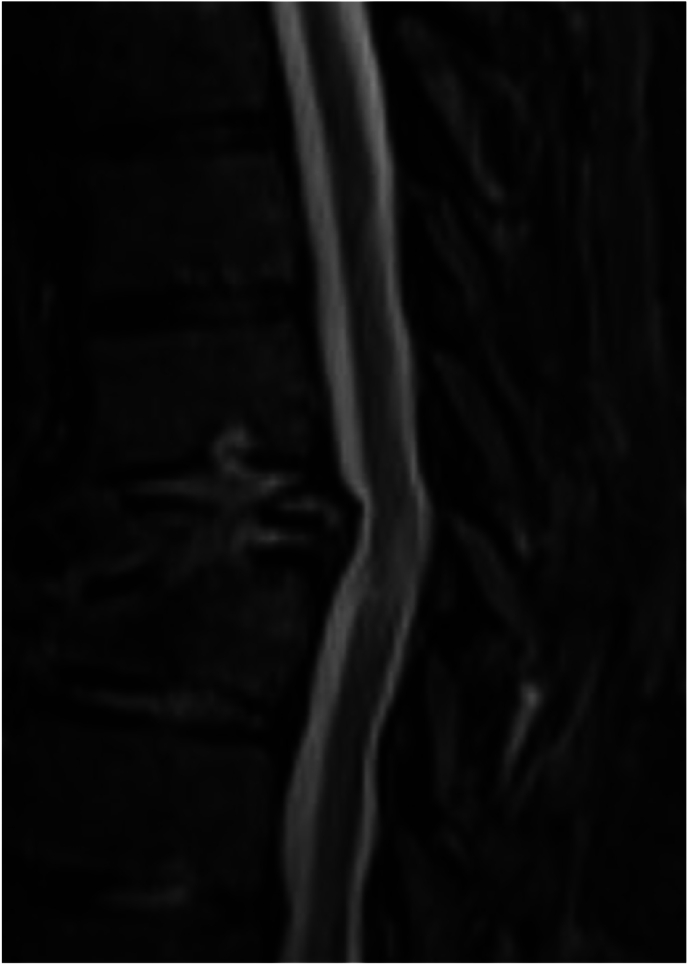
Six-month follow-up MRI thoracic spine shows a further collapse of T8 resulting in vertebral plana with new anterior aspect T9 compression fracture. Overall, improved central canal stenosis and cord signal changes with remains of retropulsed bone at T8.

After medical stabilization, the patient was transferred from the medical intensive care unit to the spinal cord injury rehabilitation unit. After six months, the patient progressed to T7 AIS D with therapy and medical management. The patient had 1/5 motor strength with bilateral plantar flexion, dorsiflexion, and great toe extension, and 3/5 strength with hip flexion and knee extension. A follow-up MRI six months after ASAS showed almost complete resolution of cord signal abnormalities and vertebral body edema, with mild central canal stenosis and persistent T8 retropulsion extending into the central canal with focal myelomalacia [Fig. 5]. The patient was discharged home at maximal assistance for activities of daily living and received all necessary adaptive equipment to meet functional needs.

## 3 Discussion

We report a rare case of a patient who experienced ASAS after approximately a week following kyphoplasty for a thoracic vertebral compression fracture. Before the occurrence of ASAS, the patient experienced a recurrence of severe mid-thoracic back pain that was comparable to the severity of the initial compression fracture, but did not seek medical care until symptoms of paraparesis and loss of pain and temperature sensation occurred. Patients should be educated on the potential risk for ASAS after vertebral augmentation procedure and seek immediate medical care in the setting of new neurological symptoms.

ASAS is an incomplete cord syndrome that primarily affects the anterior two-thirds of the spinal cord resulting in motor paralysis below the level of the lesion, as well as the loss of pain and temperature sensation at and below the lesion [3]. This condition is due to generalized hypoperfusion or direct occlusion of the ASA or any of its major radiculomedullary branches, resulting in ischemic infarction of the anterior two-thirds of the spinal cord [4]. This vascular complication may lead to significant functional disruption of the bilateral spinocerebellar and spinothalamic tracts responsible for transmitting pain and temperature sensation from the extremities to the brain, as well as bilateral corticospinal and corticobulbar tracts responsible for motor function [5]. The diagnosis of ASAS may be established with a detailed history and the physical exam findings discussed above. Neuroimaging with an MRI of the spine is the gold standard study [3,4]. Lastly, MR angiography or CT angiography can help identify the location of vascular pathology.

To date, three cases of ASAS following kyphoplasty or vertebroplasty have been reported in the literature. In 2010, Tsai et al. reported a case of ASAS caused by cement embolism after vertebroplasty at the T9-T10 levels, with CT revealing opacification of multiple vessels, including the paravertebral vessels, left intercostal artery, and a segment of the ASA [6]. This occlusion resulted in paraplegia and loss of sensitivity to pain and temperature immediately after the injury with preservation of deep pressure sensation and 2-point discrimination below the T10 level. In 2014, Bredow et al. presented a case of ASAS following a kyphoplasty without cement leakage. MRI did not reveal any cement in the spinal canal. Neurology initiated a 15,000 IU low molecular heparin bolus with symptoms resolving in 6 ​h [7]. The authors in this study did not report or hypothesize a potential mechanism for this complication, instead stating, “MRI excluded potential causes such as cement in the spinal cord, intraspinal hematoma, incorrect transpedicular approach, or myelopathy.” In 2011, Yazbeck et al. presented a case of ASAS caused by cement embolism at the segment of the ASA at T10-L1 after percutaneous vertebroplasty [8].

Compared to these three cases, our patient presented with several similarities, including clinical presentation and imaging findings consistent with ASAS. However, the aforementioned cases were caused by cement embolism with a quicker onset post-procedure. Another rare and similar clinical presentation to cement embolism is called fibrocartilaginous embolism (FCE). This presents with severe spine pain, anterior spinal artery syndrome symptoms, followed by radiological evidence of an intervertebral disc adjacent to the spinal cord infarction. The theory behind FCE is that nucleus pulposus material enters the vascular system following a traumatic event from increased intra-disc pressure by axial loading forces on the spine [9]. FCE is a diagnosis of exclusion and can only be confirmed with a biopsy for histopathological analysis at autopsy.

This case exhibited symptoms nine days after the procedure with a plausible explanation of retropulsed bone fragments from T8 vertebral body refracture based on imaging. The authors of this study acknowledge that it is impossible to rule out cement embolism or FCE as possible etiologies for this complication without a computed tomography angiogram. A paper by Lai and his colleagues looking at bone cement's chemical and physical properties suggests the average polymerization time is approximately 2–5 minutes, depending on temperature, but once the polymerization ends, the cement becomes solidified [11]. Thus, the likelihood of cement embolism nine days from the procedure is exceedingly low.

An explanation for this current case may be attributed to the mechanical retropulsion of bone fragments from the T8 vertebral body herniating into the spinal cord. In 2021, Aalbers et al. presented a paper on thoracic disc herniation contributing to spinal cord ischemia through direct compression of the anterior spinal artery or the network of radicular arteries supplying the spinal artery [10]. The anterior spinal artery runs along the anterior surface of the spinal cord; therefore, it is theorized that the mechanical forces of a herniated disc can cause extraluminal compression of the vessel resulting in cord ischemia. A systemic review on ASAS due to disc herniation by Islam and his colleagues suggests a similar mechanism, proposing that disc herniation is more likely to compress the anterior spinal medullary or radicular arteries feeding the anterior spinal artery by way of the typical posterolaterally disc displaced pathway [12]. In our current case, we hypothesize that on day eight post-kyphoplasty, the patient was experiencing an acute exacerbation of thoracolumbar pain from T8 bone fragments into the spinal cord from some exertional movement or abnormal physical activity. On day nine, the physical exam findings, clinical presentation, and MRI findings were consistent with a diagnosis of ASAS. Of note, Rollinghoff et al. published a paper on a consensus of 160 expert practitioners looking at indications and contraindications for cement augmentation procedures in OVCF and discovered that 72% of experts considered a vertebral body height collapse of greater than 70% as a relative contraindication, also known as vertebra plana [13]. American Academy of Orthopedic Surgeons and Society of Interventional Radiology guidelines suggest this is a relative contraindication for augmentation procedure due to treatment of these fractures being more technically challenging and often associated with increased rates of complication [14]. Thus, we predict this patient was at high risk for a complication based on imaging findings before kyphoplasty.

**Fig. 6:**
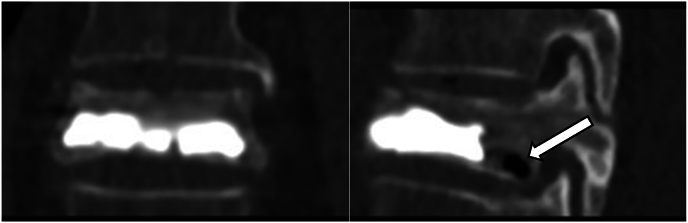
Post-kyphoplasty with evidence of intervertebral cleft and non-contact bone cement at the vertebral end plate.

Bone cement displacement is a rare complication of vertebral augmentation with an overall incidence of 2.3% based on a large single-center study by Qi et al. [15] Bone cement displacement has been described in the literature, and the most recent research suggests risk factors symptomatic bone cement displacement include anterior leakage, intravertebral vacuum cleft sign (IVC), bone cement distribution score, and paraspinal muscle degeneration [15]. The imaging from this case report supports bone fragment displacement from a refracture of T8 rather than bone cement displacement as the primary contributor to this acute myelopathy. Lee et al. report predisposing factors of refractures include intravertebral cleft (osteonecrosis) and non-polymethylmethacrylate endplate contact [16]. Respectfully looking back at this case before the complication, this individual has both of these risk factors leading to a higher risk based off most recent research. [Fig. 6].

In comparison to cerebral infarctions, treatment options for spinal cord infarctions have yet to be established in the medical community, where the guidelines for cerebral infarction management post-inciting event are well established. Currently, there is no good evidence of effective treatment options for ischemic spinal cord injuries, in part by the lack of high-quality studies. In a recent meta-analysis by Naik et al. looking at treatments for non-iatrogenic spinal cord infarctions, lumbar cerebrospinal fluid (CSF) drainage showed a significant improvement among this patient population. In contrast, other treatment options such as corticosteroids, anticoagulation, and thrombolytics showed no improvement or worsened outcomes [17]. CSF drainage has been widely studied in the cardiothoracic surgery field for the utilization of prevention of spinal cord ischemia, but little has been studied outside of retrospective case studies on this treatment modality.

Limitations of this study are the lack of angiogram and CT spine during the initial presentation of this event. MR or CT angiograms are ideal imaging techniques for localizing and classifying spinal vascular lesions. Although the classic clinical presentation and T2 weighted MRI findings of hyperintensity within the intramedullary regions are sufficient for diagnosing ASAS, the etiology remains unclear, and identifying the potential vessel or vessels contributing to ischemia can change the treatment plan. CT angiogram after kyphoplasty can effectively rule out cement emboli or bone extravasation as potential causes of ongoing ischemia. In retrospect, although the patient was experiencing acute kidney injury and rapid onset of ASAS, an intravenous fluid bolus may have been considered to permit the utilization of contrast to better visualize vessels under CT angiography and to rule out embolism as a potential cause.

## 4 Conclusion

Percutaneous kyphoplasty is an effective procedure for managing OVCF; it is not without risk for complications. This study highlights a rare instance of delayed-onset ASAS following T8 kyphoplasty with a suspected etiology of vertebral body retropulsion. Clinicians who suspect ASAS or acute myelopathy after kyphoplasty should consider obtaining a CT angiogram to identify the occluded or compressed vessel leading to ischemia and rule out cement embolism.

## Financial disclosures

None of the authors involved in the creation of this case report have identified any competing interests and have no sources of funding to declare for this manuscript.

## Informed consent

Consent was obtained by the participant in this case report.

## Declaration of competing interest

The authors declare that they have no known competing financial interests or personal relationships that could have appeared to influence the work reported in this paper.
